# Genetic Transformation of Recalcitrant Upland Switchgrass Using Morphogenic Genes

**DOI:** 10.3389/fpls.2021.781565

**Published:** 2022-02-08

**Authors:** Nuoya Xu, Minjeong Kang, Jacob D. Zobrist, Kan Wang, Shui-zhang Fei

**Affiliations:** ^1^Department of Horticulture, Iowa State University, Ames, IA, United States; ^2^Crop Bioengineering Center, Iowa State University, Ames, IA, United States; ^3^Interdepartmental Plant Biology Major, Iowa State University, Ames, IA, United States; ^4^Department of Agronomy, Iowa State University, Ames, IA, United States; ^5^Interdepartmental Genetics and Genomics Major, Iowa State University, Ames, IA, United States

**Keywords:** *Agrobacterium*-mediated, auxotrophic, *Baby boom*, Cre-Lox, immature leaf segments, *Panicum virgatum*, seedling-derived, *Wuschel2*

## Abstract

Switchgrass (*Panicum virgatum*) is an excellent feedstock for biofuel production. While genetic transformation is routinely done in lowland switchgrass, upland cultivars remain recalcitrant to genetic transformation. Here we report the establishment of an efficient and reproducible transformation protocol for two upland cultivars, ‘Summer’ and ‘Blackwell’, by ectopic overexpression of morphogenic genes, *Baby boom* (*Bbm*) and *Wuschel2* (*Wus2*). Two auxotrophic *Agrobacterium* strains, LBA4404Thy- and EHA105Thy-, each harboring the same construct containing *ZmBbm*, *ZmWus2*, and a green fluorescence protein (GFP) gene, *ZsGreen1*, were used to infect immature leaf segments derived from *in vitro* grown seedlings. The *Agrobacterium* strains also contain a transformation helper plasmid that carry additional copies of *Agrobacterium* virulence genes. GFP-expressing calli were identified and selected for regeneration. The highest transformation efficiency of 6% was obtained for the tetraploid cultivar Summer when LBA4404Thy- was used for infection, which is twice of that for the octoploid cultivar Blackwell. LBA4404Thy- consistently outperformed EHA105Thy- on transformation frequency across the two cultivars. Fifteen randomly selected putative transgenic plants of Summer and Blackwell, representing independent callus events, were confirmed as transgenic by the presence of the transgene, *ZmAls*, and the absence of *AtuFtsZ*, a chromosomal gene specific to the *Agrobacterium* strain LBA4404 using polymerase chain reaction. Transgene integration and expression was further confirmed by the detection of GFP in roots, and the resistance to herbicide injury to leaves of selected putative transgenic plants. The *ZmBbm* and *ZmWus2* genes were successfully removed from 40 to 33.3% of the transgenic plants of Summer and Blackwell, respectively, *via* the Cre-Lox recombination system upon heat treatment of GFP-expressing embryogenic calli. Our successful transformation of recalcitrant upland switchgrass provides a method for gene function analysis and germplasm enhancement *via* biotechnology.

## Introduction

Switchgrass (*Panicum virgatum*) is a C4 perennial grass native to the United States. Because of its high biomass yield potential, low inputs, ability to grow well on marginal soils and well-established agronomic practices, it has become an important factor in US energy strategy. In 1991, switchgrass was selected by the United States Department of Energy as an herbaceous model species for the production of bioenergy (McLaughlin et al., 2002; McLaughlin and Kszos, 2005). It is a valuable forage grass and an excellent choice for soil conservation due to its extensive fibrous root system (Vogel et al., 2011; Mitchell and Schmer, 2012; Schmer et al., 2014; Aurangzaib et al., 2016). There are two major ecotypes of switchgrass, the lowland ecotype with thick stems and a predominantly bunch type growth habit that is adapted to warmer regions, and the upland ecotype with finer stems and highly developed rhizomes, adapted to more temperate regions (Vogel et al., 1985). Lowland switchgrass cultivars are high yielding, but lack adequate winter hardiness. In contrast, upland cultivars are winter hardy and well-adapted to northern climates (Casler et al., 2011). Lowland cultivars are predominantly tetraploid whereas upland cultivars are tetraploid or octoploid while aneuploids are also widespread (Costich et al., 2010). Switchgrass is open-pollinated due to its self-incompatibility; as such, commercially available cultivars are typically synthetic cultivars that are highly heterozygous and heterogeneous (Martinez-Reyna and Vogel, 2002). Lowland and upland cultivars of the same ploidy can readily hybridize, suggesting that divergence between the two occurred recently (Casler, 2012).

Considerable genomic resources have been developed for switchgrass. This includes numerous molecular markers of various types (Missaoui et al., 2005; Cortese et al., 2010), genetic maps and QTLs mapped for important traits (Dong et al., 2015; Tornqvist et al., 2018), gene expression profiles (Palmer et al., 2015, 2017) and a reference genome sequence assembly of the lowland switchgrass cultivar, ‘Alamo’, an allotetraploid (2n = 4x = 36) with two subgenomes, N and K. In addition, robust genetic transformation protocols have been established for lowland cultivars (Li and Qu, 2011; Fu et al., 2012; Liu et al., 2018, 2020; Qiao et al., 2021). Despite the significant progresses, the functions of most switchgrass genes remain largely uncharacterized, particularly in upland switchgrass cultivars which are known for their recalcitrance to genetic transformation (Song et al., 2012; Merrick and Fei, 2015; Lin et al., 2017). Attempts to transform upland switchgrass have only met with very limited success. Liu et al. (2015) reported a significant improvement on shoot regeneration in upland switchgrass with the identification of “shell-core” callus structure and the development of type II callus by isolating and culturing the pre-embryogenic callus “core.” Successful transformation of an upland cultivar ‘Dacotah’ was reported in their study; however, despite the significantly improved plant regeneration efficiency for the cultivar Blackwell, there was no mention of a successful transformation of this cultivar (Liu et al., 2015). Ogawa et al. (2016) reported the successful transformation of an upland cultivar ‘Trailblazer’ by optimizing the cocultivation and preculture conditions of type I callus, but failed to transform the cultivar Blackwell. Both reports used caryopsis-derived callus as explants for transformation which affects the reproducibility of the developed protocols because each caryopsis represents a distinct genotype in synthetic cultivars. The lack of transformation protocol in upland switchgrass cultivars severely hampers the effort on leveraging available genomic resources for switchgrass cultivar improvement. The preferred method of genome editing, CRISPR-Cas9, requires a reliable genetic transformation protocol to implement gene editing and it has not been reported in upland switchgrass (Jinek et al., 2012; Hsu et al., 2014).

Recalcitrance to genetic transformation is primarily caused by the inability of regenerating functional plants from an explant cultured *in vitro*, *via* either *de novo* organogenesis or somatic embryogenesis. The ability to regenerate varies considerably between species and even between cultivars/genotypes within a species. This is likely the result of genetic variation (Xu and Huang, 2014; Lardon and Geelen, 2020) and the culture environment including the chemical composition of a culture medium (Fei et al., 2002; Lin et al., 2017), culture conditions such as temperature, photoperiod and type of explant (Xi et al., 2009; Merrick and Fei, 2015). Lardon and Geelen (2020) grouped genetic factors underlying shoot regeneration ability into (1) master regulators, i.e., developmental regulators or morphogenic genes such as *Wuschel* genes that are conserved among species and (2) conditional regulators whose effect are influenced by the type of explants and culture conditions. *Wuschel2* (*Wus2*), a homeodomain-containing transcription factor gene and *Baby boom* (*Bbm*) gene which encodes an AP2/ERF transcription factor are among the several genes that significantly affect morphogenesis. Both *Bbm* and *Wus2* have been shown to promote somatic embryogenesis in several species (Zuo et al., 2002; Kulinska-Lukaszek et al., 2012; Conner et al., 2015). Lowe et al. (2016) demonstrated that by overexpression of *Bbm* and *Wus2*, transgenic monocots plants were successfully recovered in genotypes or explant types that were otherwise recalcitrant to genetic transformation. Ectopic expressing of the maize *Bbm* and *Wus2* genes in sugarcane or sorghum also had a similar effect (Lowe et al., 2016; Mookkan et al., 2017; Nelson-Vasilchik et al., 2018).

While the morphogenic genes have greatly improved maize transformation process, it had been shown that the *Bbm* and *Wus2* genes had pleiotropic effects on plant growth and development. Their overexpression could lead to developmental abnormality and infertility (Lowe et al., 2018). To overcome these issues, the Cre-Lox recombination system (Zhang et al., 2003; Chong-Pérez et al., 2012) can be employed to remove the *Bbm* and *Wus2* genes before regeneration takes place. Lowe et al. (2016) described a method in which the T-DNA contained the CRE recombinase gene driven by rab17, a drought inducible promoter. When transgenic tissues were subjected to desiccation, the *Bbm* and *Wus2* genes, flanked by two *loxP* sites, could be successfully excised, making transgenic plants free of morphogenic genes.

The goal of our research was to develop an efficient and reproducible transformation for two upland switchgrass cultivars, Summer, a tetraploid and Blackwell, an octoploid, both representative of the ploidy levels commonly observed in upland switchgrass by overexpressing the morphogenic genes *ZmBbm* and *ZmWus2* from maize. The specific objectives were to (1) study the effect of cultivar and *Agrobacterium* strains on overall transformation frequency using immature leaf segments derived from *in vitro* grown seedlings; (2) determine the efficiency of excising the morphogenic genes with the heat inducible Cre-Lox recombination system. We show that the explants infected by *Agrobacterium* strains carrying the morphogenic genes can produce embryogenic callus pieces that generated transgenic plants. Further, the Cre-Lox recombination system was successfully implemented to remove the morphogenic genes from the transgenic plants upon heat treatment of GFP-expressing embryogenic calli. Our results demonstrate that the strategy of deployment and removal of morphogenic genes has led to transformation of recalcitrant upland switchgrass genotypes, which were not possible when using conventional transformation approaches.

## Materials and Methods

### Plant Materials

Two upland switchgrass cultivars, Summer, a tetraploid and Blackwell, an octoploid were used for the present study (Alderson and Sharp, 1994). Seeds were kindly provided by Dr. Serge Edmé at United States Department of Agriculture-Agricultural Research Service. Greenhouse conditions for growing upland switchgrass plants are set at a 25°C, 16/8 h (day/night) photoperiod and a light intensity of approximately 400 μM/m^2^/s. Plants are typically grown in plastic pots with a diameter of 15 cm and were fertilized with Peters Cal Mag special 15-5-15 at 150 ppm N every 2 weeks.

### Morphogenic Gene Construct and *Agrobacterium* Strains

**FIGURE 1:**
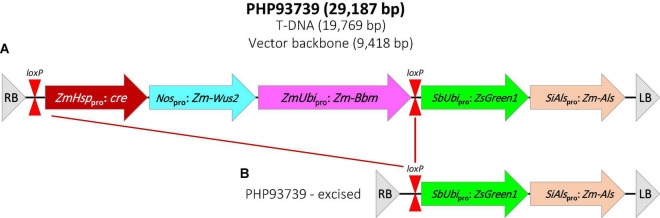
Schematic illustration of the construct PHP93937 used for all transformation experiments. **(A)** T-DNA region of PHP93739. RB, T-DNA right border; *loxP*, CRE recombinase target site; *ZmHsp*_pro_: *cre*, maize heat shock protein 17.7 promoter (*Zm-Hsp17.7*) + *cre* gene + potato proteinase inhibitor II (*pinII*); *Nos*_pro_: *Zm-Wus2*, *Agrobacterium* nopaline synthase promoter (*Nos*) + maize *Wuschel2* gene (*Zm-Wus2*) + maize *In2-1* terminator; *ZmUbi*_pro_: *Zm-Bbm*, maize ubiquitin promoter/intron (*ZmUbi*) + maize *Baby boom* gene (*Zm-Bbm*) + maize ubiquitin terminator (*Zm-Ubi*); *SbUbi*_pro_: *ZsGreen1*, sorghym ubiquitin promoter/intron (*SbUbi*) + green fluorescent protein *ZsGreen1* gene + rice ubiquitin terminator (*OsUbi*); *SiAls*_pro_: *Zm-Als*, *Setaria italica* acetolactase synthase (*SiAls*) promoter + maize Als (*Zm-Als*) gene + sorghum ubiquitin terminator (*Sb-Ubi*); LB, T-DNA left border. **(B)** T-DNA region in transgenic plant after the heat-treatment to remove the morphogenic genes and *cre* gene.

Transformation construct PHP93739 was kindly provided by Dr. William Gordon-Kamm at Corteva Agriscience. The T-DNA region of PHP93739 contains five expression cassettes (Figure 1A). The first one contains a maize Hsp17.7 promoter driving the *cre* gene; the second one contains the *Agrobacterium nos* promoter driving the maize *Wus2* gene; the third contains a maize ubiquitin promoter driving the maize *Bbm* gene; the fourth cassette contains a sorghum ubiquitin promoter driving the *ZsGreen1* gene and the fifth cassette contains the *Setaria italica* acetolactate synthase (*SiAls*) promoter driving the mutated maize *Als* gene. Cassettes 1–3 are flanked by two *loxP* sites.

A thymidine auxotrophic (Thy-) version of the *Agrobacterium* strains of LBA4404 (Lowe et al., 2016; kindly provided by Dr. William Gordon-Kamm of Corteva Agriscience) and EHA105 (Aliu et al., 2020) were used to harbor the binary vector construct PHP93739. Both *Agrobacterium* strains contain the ternary helper plasmid PHP71539 to increase virulence (Anand et al., 2018; kindly provided by Dr. William Gordon-Kamm of Corteva Agriscience).

### Explant Preparation

**FIGURE 2:**
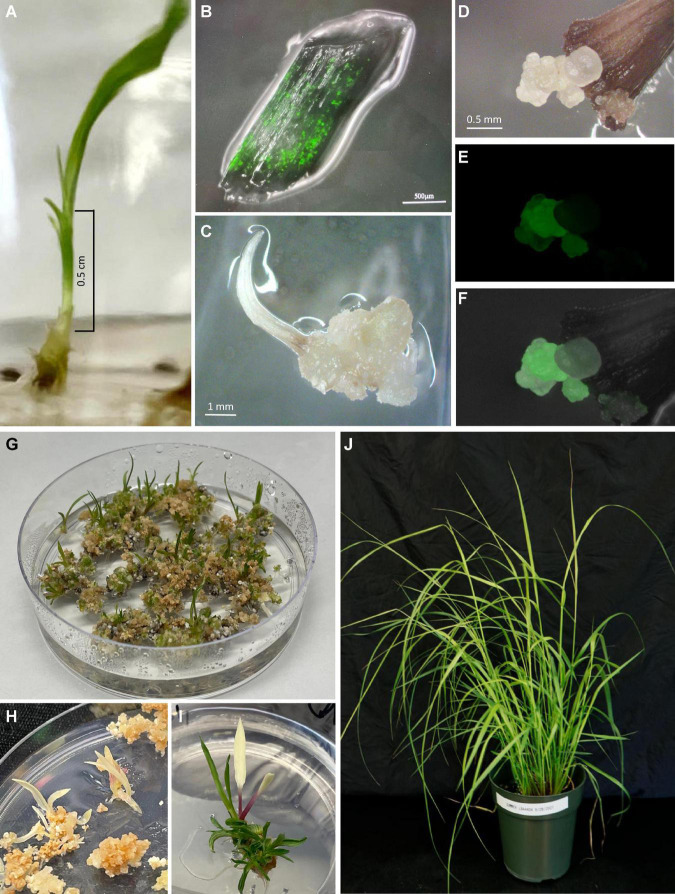
Regeneration of transgenic plants of the upland switchgrass cultivar Summer. **(A)** 7-day old seedlings from which basal stems (within the bracket) containing whorls of leaves including leaf sheaths and blades were excised for *Agrobacterium* infection. **(B)** Transient expression of GFP on immature leaf segments 7 days post-inoculation. **(C)** Non-embryogenic callus formed on explants treated with RM medium only (no-infection control). **(D)** Embryogenic callus formed on explants infected with *Agrobacterium*. **(E)** GFP expression on the same callus as shown in panel **D**. **(F)** Overlay of images **(D,E)**. **(G)** Regeneration of healthy shoots 3 weeks following the heat-shock treatment. **(H)** An embryogenic callus producing entirely albino shoots. **(I)** An embryogenic callus producing a mixture of green shoots and an albino shoot. **(J)** Soil-grown transgenic plants 2 months after being established in the greenhouse.

Caryopses of the two cultivars were sterilized by first rinsing them in 75% ethanol for 1 min, followed by disinfection with undiluted commercial bleach (5.25% sodium hypochlorite, NaHClO_3_) plus 0.1% Tween ^®^ 20 for 2 h. Sterilized caryopses were rinsed in autoclaved distilled deionized water 3–4 times, each ∼5 min before cultured on Seed Germination Medium (SG/RTM, Table 1). Caryopsis culture took place in a biological incubator with a photoperiod of 16/8 h (light/dark) and a light intensity of 140 μM/m^2^/s and a temperature of 25°C. Basal part of seedlings (6–8 mm long) were aseptically excised from either the main stem or tillers of 10- to 14-day-old seedlings grown *in vitro*. These basal seedling segments were comprised of whorled immature leaf segments which were cut into segments of 3–4 mm long (Figure 2A; Denchev and Conger, 1994). Whorled immature leaf segments were carefully separated from each other and used for subsequent *Agrobacterium* infection.

**TABLE 1:** Composition and preparation of media used in the experiments (modified based on Li et al., 2015).

Medium	Composition and preparation
YP	10 g/L NaCl, 5 g/L yeast extract, 10 g/L peptone, 15 g/L Bacto™ agar, pH 7.0; autoclave; cool down to 55°C; add 50 mg/L thymidine, 50 mg/L spectinomycin, 50 mg/L gentamicin, all filter-sterilized.
Resuspension Medium (RM)	10 mL/L MS major salts stock^1^, 1 mL/L MS minor salts stock^2^, 0.5 mL/L MS FeSO_4_/EDTA stock^3^, 5 mL/L MS vitamins stock^4^, 30 g/L sucrose, pH 5.4; autoclave; cool down to 55°C; add 4 mg/L 2,4-D, 50 mg/L thymidine, 100 μM acetosyringone, all filter-sterilized.
Co-cultivation Medium (CCM)	10 mL/L MS major salts stock^1^, 1 mL/L MS minor salts stock^2^, 0.5 mL/L MS FeSO_4_/EDTA stock^3^, 5 mL/L MS vitamins stock^4^, 30 g/L maltose, pH 5.4, 6 g/L Phytagel™ autoclave; cool down to 55°C; add 4 mg/L 2, 4-D, 0.8 mg/L BAP, 50 mg/L thymidine, 100 μM acetosyringone, all filter-sterilized.
Callus Induction Medium (CIM)	MS basal medium with 30 g/L maltose, pH 5.8, 6 g/L Phytagel™, autoclave; cool down to 55°C; add 4 mg/L 2,4-D, 0.8 mg/L BAP, 2 mg/L L-proline, 150 mg/L timentin, 100 μM acetosyringone (optional), all filter-sterilized.
Shoot Induction Medium (SIM)	MS basal medium with 30 g/L maltose, 3 g/L Phytagel™ pH 5.8; autoclave; cool down to 55°C; add filter-sterilized BAP at 0.5 mg/L.
Seed Germination/Rooting Medium (SG/RTM)	MS basal medium with 30 g/L maltose, pH 5.8, 3 g/L Phytagel™, autoclave; cool down to 55°C.

*^1^ MS major salts stock (10×): 19 g/L KNO_3_, 16.5 g/L NH_4_NO_3_, 4.4 g/L CaCl_2_.2H_2_O, 3.7 g/L MgSO_4_.7H_2_O, 1.7 g/L KH_2_PO_4_.*

*^2^ MS minor salts stock (100×): 620 mg/L H_3_BO_3_, 2.5 mg/L CoCl_2_.6H_2_O, 2.5 mg/L CuSO_4_.5H_2_O, 2230 mg/L MnSO_4_.4H_2_O, 83 mg/L KI, 25 mg/L Na2MoO_4_.2H_2_O, 860 mg/L ZnSO_4_.7H_2_O.*

*^3^ MS FeSO_4_/EDTA stock (100×): 2780 mg/L FeSO_4_.7H_2_O, 3670 mg/L FeNaEDTA.*

*^4^ MS vitamins stock (200×): 400 mg/L glycine, 20 g/L myo-inositol, 100 mg/L nicotinic acid, 100 mg/L pyridoxine HCl, 10 mg/L thiamine.*

### *Agrobacterium* Infection, Callus Induction, and Plant Regeneration

The overall *Agrobacterium*-mediated infection and regeneration process was based on Li et al. (2015) with modifications. Briefly, *Agrobacterium* cultured on solid YP medium (Table 1) for 18–24 h in 28^°^C before they were collected with inoculating loops and dispensed into 15 mL of the Resuspension Medium (RM, Table 1) in a 50 mL tube. The *Agrobacterium* suspension was measured using a spectrophotometer and adjusted to optical density (OD) at the wavelength of 650 nm into desired densities with the RM.

One hundred immature leaf segments of 3–4 mm long dissected from *in vitro* grown switchgrass seedlings described above were submerged in a small Petri plate (60 mm diameter) containing 10 mL of the *Agrobacterium* suspension. The plate was placed on a shaker (120 rpm) for 10 min at the room temperature (∼25^°^C). Following infection, immature leaf segments were transferred to a plate containing the Cocultivation Medium (CCM, Table 1) for 3 days at 26°C in the dark, before they were transferred to Callus Induction Medium (CIM, Table 1) at 20 immature leaf segments per plate. Explants were subcultured twice, with a 4-week interval, on CIM without selection.

Heat treatment to remove the morphogenic genes on the T-DNA was performed on GFP-expressing embryogenic calli as described (Masters et al., 2020). Briefly, CIM plates with callus segments were placed in a clean plastic shoebox (15.24 cm × 21.59 cm × 7.62 cm) with three layers of moistened paper towels. The plates were placed at 45^°^C with ∼70% relative humidity for 2 h. Heat-treated calli were placed at 26^°^C in the dark for 1 h of resting. These calli were then cultured in the Shoot Induction Medium (SIM, Table 1) without selection and placed in a biological incubator with a photoperiod of 16/8 h light/dark and a light intensity of 140 μM/m^2^/s at 25°C.

Shoots of 0.5 cm or longer were transferred to Rooting Medium (SG/RTM, Table 1) under the same culture condition as for shoot induction. Rooted plantlets were transplanted into 4-inch plastic pots containing a commercial soil mix (Sunshine soil mix #1, Sun Gro) of peat moss and perlite and were moved to a mist room for acclimation for 7–10 days, and subsequently moved to a greenhouse with conditions described above.

### Characterization of Transgenic Plants

DNA was extracted from leaf tissues of greenhouse-established putative transgenic plants using the CTAB method with minor modifications (Liu et al., 2018). To detect the presence of the herbicide resistance gene, *ZmAls*, a pair of forward primer ALS1-F (5′ ACCATCAACCTGTGCGTGAT 3′) and reverse primer ALS1-R (5′ ACTTCTGGTTCTTGGCGTCG 3′) was used for PCR amplification. The PCR conditions were 2 min of the initial denaturation at 95°C, 34 cycles of the following: 1 min of denaturation at 95°C, 1 min of annealing at 63°C for *ZmAls* and 58°C for *cre*, respectively, and 1 min extension at 72°C. The final extension was 5 min at 72°C. PCR amplicons were visualized *via* SYBER Safe-based gel electrophoresis. To determine whether the morphogenic genes were removed from transgenic plants, the *cre* gene which is tightly linked to the *ZmBbm* and *ZmWus2* genes and flanked by the *loxP* sites was amplified *via* PCR using the forward primer CRE1-F (5′ GAAGACTAGAACCGAACC 3′) and CRE1-R (5′ GGCATCACCATGTTTTGG 3′). The PCR conditions for the *cre* gene was identical to that for the *ZmAls* gene except for the annealing temperature which was 57°C.

To exclude the possibility of *Agrobacterium* persisting in intercellular spaces in putative transgenic plants that could have resulted in false positives in previous PCR reactions, reverse transcription (RT)-PCR was conducted for the *ZsGreen1* gene. RNA isolation was done with TRIzol from actively growing leaf tissues (∼70 mg) according to the manufacturer’s protocol. First-strand cDNA synthesis was done with the Maxima H Minus First Strand cDNA Synthesis Kit, with dsDNase (ThermalFisher Scientific). *ZsGreen1* was amplified *via* PCR by using primer ZsGreen1-F (5′ AGATGACCA TGAAGTACCGC 3′) and ZsGreen1-R (5′ TCATCTTCTT CATCACGGGG 3′). Annealing was done at 57°C for 30 s and the PCR was conducted for 34 cycles. The switchgrass actin (*PvActC*) was amplified as an internal control with the same PCR program as used for *ZsGreen1* by using primer Act1-F (5′ CAAGATTTGGAGATCCCG 3′) and Act1-R (5′ AATGCTCCACGGCGAACA 3′) (Xu et al., 2018).

To further verify that Agrobacteria were no longer present in greenhouse-established plants, PCR amplification was done for a strain-specific chromosomal gene, *AtuFtsZ* for LBA4404 (Deeba et al., 2014) in a subset of DNA samples used for PCR amplification of the *ZmAls* described above. The positive control was obtained by amplifying chromosomal DNA isolated from LBA4404Thy- The primer FtsZ -F (5′ GAACTTACAGGCGGGCTGGGT 3′) and FtsZ -R (5′ CGCCGTCTTCAGGGCACTTTCA 3′) were used for PCR for 34 cycles with an annealing temperature of 62°C for 30 s. GoTaq ^®^ Green Master Mix was used for all PCR and RT-PCR reactions.

In addition to PCR verifications, fluorescence microscopy was conducted to detect GFP signals in root tissues of select putative transgenic plants. Root tissues were collected from 6-week-old greenhouse grown wild type and putative transgenic plants of upland switchgrass cultivar Summer. Roots were washed with tap water and were cut into 0.5 cm segments. Root segments were placed on glass slides without cover slips and observed under a Zeiss Axiostar Plus Binocular microscope or an Olympus SZH10 stereo microscope equipped with a GFP filter.

Herbicide painting experiment was conducted to examine leaf response to herbicide applications. Newly expanded young leaves of wild type and putative transgenic plants of Summer and Blackwell were painted with herbicide Aligare Mojave 70 EG (active ingredient imazapyr at 7.78%) with a concentration of 0.9 g/mL by using a Q-tip. Herbicide was applied to both adaxial and abaxial surface of the upper half of each leaf. Painting was applied twice with the second application performed 5 days after the initial painting. Results were collected 7 days after initial painting.

## Results

### The Effect of *Agrobacterium* Strain and Switchgrass Cultivar on Callus Induction

To determine the effect of *Agrobacterium* strains and switchgrass cultivars on transformation frequency, we infected 100 immature leaf segments (20 leaf segments per petri dish) collected from each of the two cultivars, Summer and Blackwell, with either LBA4404Thy- or EHA105Thy- at the optical density (OD_650_) of 0.35–0.45 measured at the wavelength of 650 nm. Both strains contained PHP93739 (Figure 1A). The infection experiments was repeated five times, resulting in a total of 500 explants for each treatment. For the no Agro infection control, 30 immature leaf segments were treated with RM (Table 1) for each replication and with five replications, a total of 150 explants were treated. Initial examination of GFP signals on immature leaf segments was performed about 5–7 days after cocultivation with the two *Agrobacterium* strains LBA4404Thy- and EHA105Thy-. For immature leaf segments in which GFP signals were detected, strong GFP signals were typically detected across the entire leaf segments (Figure 2B).

Calli started to form about 2 weeks after co-cultivation and continued to develop for both Summer and Blackwell regardless of the *Agrobacterium* strains used for infection. Callus formation was also observed in immature leaf segments that were not infected by either *Agrobacterium* strains (Figure 2C). However, embryogenic callus, as defined by their morphology and growth rate that are characteristic of the type II callus in other Poaceae species were found only on immature leaf segments infected with *Agrobacterium* containing the morphogenic genes. These calli typically have creamy, white color and nodular structures with strong GFP signals (Figures 2D–F). At the end of the second subculture, data on the production of callus and embryogenic callus were collected and analyzed. As summarized in Table 2, for the no infection control, the percentage of callus induction was 15.3% for both Summer and Blackwell, but none of the calli formed on immature leaf segments from the control assumed the appearance of embryogenic callus. For the tetraploid cultivar Summer, infection with the LBA4404Thy- resulted in a callus induction frequency of 14.4% of which 7.6% were GFP-expressing embryogenic calli. In contrast, infection with the EHA105Thy- resulted in an induction frequency of 7.8% of which only 1.4% were GFP-expressing embryogenic calli. For the octoploid cultivar Blackwell, infection with the LBA4404Thy- resulted in a callus induction frequency of 11.6% of which 4.8% were GFP-expressing embryogenic calli. Infection of Blackwell with the EHA105Thy- resulted in a callus induction frequency of 5.4% of which only 1.0% were GFP-expressing embryogenic calli (Table 2).

**TABLE 2:** Summary of transformation experiments using two *Agrobacterium* strains.

Cultivar	*Agrobacterium* strains^1^	Total # leaf segments infected^2^	% explants formed callus	% GFP(+) EC^3^	# shoots/GFP(+) EC^3^	% GFP(+) EC produced albino^3^	Overall transformation frequency (%)^4^
Summer	No infection	150	15.3 ± 6.1	0.0	0.0	0.0	0.0
	LBA4404THY-	500	14.4 ± 5.6	7.6 ± 3.6	5.3 ± 1.9	17.5 ± 19.5	6.0 ± 3.9
	EHA105THY-	500	7.8 ± 1.9	1.4 ± 0.9	3.8 ± 1.6	43.3 ± 36.5	0.8 ± 0.8
Blackwell	No infection	150	15.3 ± 7.0	0.0	0.0	0.0	0.0
	LBA4404THY-	500	11.6 ± 3.5	4.8 ± 2.2	1.0 ± 1.2	20.0 ± 44.7	3.0 ± 1.2
	EHA105THY-	500	5.4 ± 2.5	1.0 ± 1.2	1.2 ± 1.6	0.0	0.6 ± 0.9

*^1^Both strains carry PHP93739 (Figure 1A) and PHP71539 vir helper plasmid (Anand et al., 2018).*

*^2^Data from five independent infection experiments (100× leaf segments per infection) for each strain.*

*^3^EC, embryogenic callus; Mean percentage ± Standard Deviation from five independent infection experiments for each strain.*

*^4^Transformation frequency defined as the percentage of rooted shoots of total infected immature leaf segments. Mean percentage ± Standard Deviation from five independent infection experiments for each strain.*

### Excision of Morphogenic Genes and Regeneration of Transgenic Plants

The GFP-expressing embryogenic calli were further subcultured to increase size and vigor. To remove the morphogenic genes in transformed tissues, the transformation construct PHP93739 carried an inducible Cre-Lox recombination system. As shown in Figure 1A, the *cre* recombinase gene used in the present study was driven by the maize heat shock protein 17.7 promoter (*Zm-Hsp17.7*). All GFP-expressing calli of a diameter 1.0 cm or larger were heat treated for 2 h (Masters et al., 2020) prior to transferring to shoot regeneration medium. The heat-activated CRE recombinase would cleave the *loxP* sites and remove both the *cre* gene cassette and the morphogenic genes (Figure 1B). Shoots started to appear as soon as 2 weeks after the transfer, but most shoot formation occurred between 3 and 5 weeks following the transfer to the regeneration medium (Figure 2G).

**FIGURE 3:**
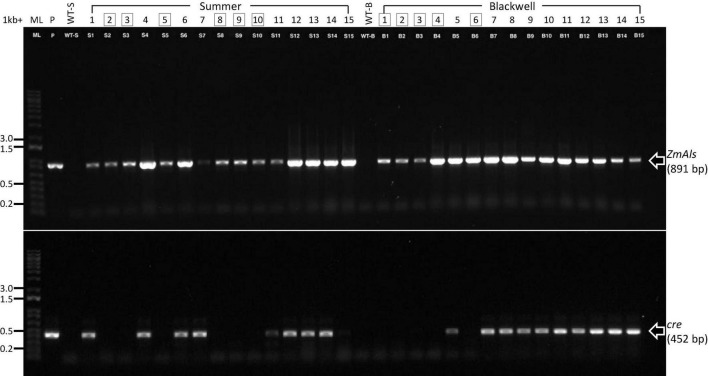
Characterization of putative transgenic plants. PCR reactions were performed for 15 putative transgenic plants of Summer (S1–S15) and Blackwell (B1–B15), respectively, for the presence of the herbicide resistance gene *ZmAls* (891 bp, top panel) and *cre* gene (452 bp, lower panel). ML, 1-kb molecular ladder; P, plasmid DNA as positive control; WT-S, Summer wild type; WT-B, Blackwell wild type. Boxed numbers denote desired transgenic plants with the *cre* gene excised.

PCR analysis was performed on 15 randomly selected putative transgenic T0 plants of Summer or Blackwell (Figure 2J). Figure 3 (top panel) shows that all plants contain an 891 bp fragment of the *ZmAls* transgene. These plants were also PCR tested for the presence of genes in between the *loxP* sites (Figure 1). The results showed that the *cre* gene was absent from 6 out of 15 (40%) plants for Summer and 5 out 15 (33%) plants for Blackwell (Figure 3, bottom panel), suggesting the successful removal of the transgenes, including the *ZmBbm* and *ZmWus2* genes in between the *loxP* sites in these plants.

The average number of shoots regenerated from each embryogenic callus varied considerably between cultivars and to a lesser degree between the *Agrobacterium* strains that were used for infection. The average number of shoots produced per embryogenic callus were 5.3 and 3.8 for Summer and 1.0, and 1.2 for Blackwell infected with LBA4404Thy- and EHA105Thy-, respectively (Table 2 and Figure 2G).

Albino shoots were observed from regenerants of both cultivars, with 17.5 and 43.3% of the regenerating GFP-expressing calli in Summer when infected LBA4404Thy- and EHA105Thy-, respectively (Table 2). For Blackwell, none of the regenerating GFP-expressing calli from EHA105Thy- infection produced albino shoots while 20% of the calli from the infection of LBA4404Thy- produced albino shoots (Table 2 and Figures 2H,I). No molecular analysis was performed on any of the albino plants.

**FIGURE 4:**
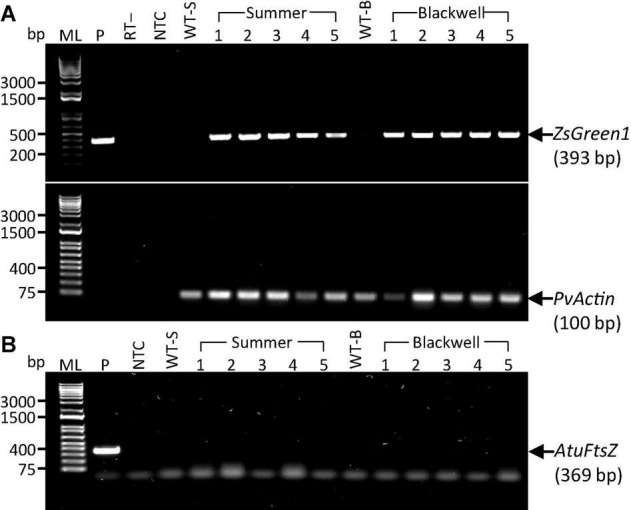
Detection of transgene expression and the presence of remnant *Agrobacterium* cells in putative transgenic plants. **(A)** RT-PCR results for the *ZsGreen1* gene with a target fragment size of 393 bp (top panel) in five putative transgenic plants of the upland switchgrass cultivars ‘Summer’ (S1–S5) and ‘Blackwell’ (B1–B5). The *PvActin* gene with a target fragment of 100 bp (bottom panel) was used as an internal control. P, plasmid DNA of PHP93739 as positive control; RT-, reverse transcriptase minus as negative control. **(B)** PCR results for the LBA4404 chromosomal gene, *AtuFtsZ*, in the same sets of plants as in panel **A**. P, the target fragment of 369 bp for the *AtuFtsZ* gene amplified with chromosomal DNA from LBA4404 as the template. Faint bands in panel B are primer dimers. ML, 1 kb molecular ladder; NTC, No template control; WT-S, Summer wild type plant; WT-B, Blackwell wild type plant. DNA samples for S1–S5 and B1–B5 were identical to S1–S5 and B1–B5 used in Figure 3.

To further confirm the transgenic nature of the T0 plants and to exclude the possibility of false PCR positive results due to the *Agrobacterium* persistence in tissue culture, we performed reverse transcription (RT)-PCR on the PCR-confirmed plants. Five plants from each cultivar, S1-S5 and B1-B5 were further tested for the presence of the *ZsGreen1* transcript by RT-PCR. All events were shown to be positive, indicating the successful integration and expression of the transgene (Figure 4A). Additional PCR test for the *Agrobacterium* strain-specific chromosomal gene, *AtuFtsZ*, failed to amplify the gene, suggesting that *Agrobacterium* cells could not be detected in plant tissues of the same selected plants shown to be positive for the transgene (Figure 4B). This result eliminates the possibility of *Agrobacterium* causing false positives in previous PCR tests. It is worth noting that auxotrophic strains were used in this work. These *Agrobacterium* strains are not able to survive in absence of thymidine. Therefore, the persistence of *Agrobacterium* in regenerated plants should be greatly reduced.

**FIGURE 5:**
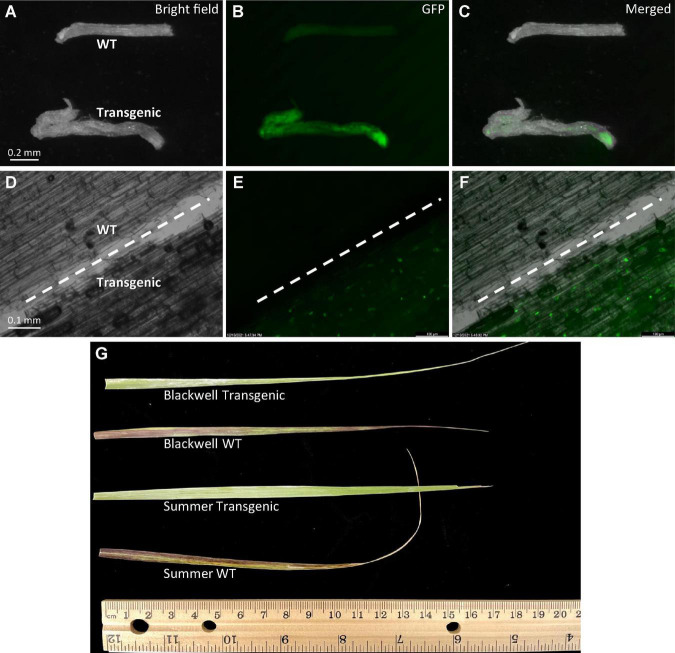
Phenotypic characterization of transgenic plants. **(A–C)** Observation of GFP signals with an Olympus florescent stereomicroscope in roots of 10-week-old transgenic plant, S8 of ‘Summer’ (Transgenic) and the wild type (WT) of similar age under either bright field **(A)**, GFP **(B)**, or merged **(C)**. **(D–F)** Observation of GFP signals with a Zeiss upright microscope in both WT and transgenic roots under either bright field **(A)**, GFP **(B)** or merged **(C)**. The white dashed line in each image separates the wild type root (upper left) from the transgenic root (lower right). **(G)** Leaf response to herbicide injury. Herbicide Alligare Mojave 70 EG (active ingredient imazapyr at 7.78%) was applied twice at a concentration of 0.9 g/mL with a 5-day interval to newly expanded leaves of the wild type and putative transgenic plants of Summer and Blackwell. Image was taken at the 7th day after the first painting.

More evidence of transgene integration and expression came from the observation of strong GFP signals in roots of selected transgenic plants of Summer whereas no GFP signals were detected in wild type plants (Figures 5A–F). Moreover, leaves from transgenic plants of both cultivars did not show herbicide injury while the wild type plants exhibited visible injuries caused by the application of imazapyr-containing herbicide (Figure 5G), further confirming the integration and expression of the transgenes.

Upon PCR verification for the presence of the *ZmAls* gene in putative transgenic plants that were established in the greenhouse, we calculated the overall transformation frequency for each treatment by the number of GFP-expressing embryogenic callus that regenerated into PCR-positive, healthy plants divided by the number of infected leaf segments. The overall transformation frequencies for the tetraploid Summer infected with LBA4404Thy- and EHA105Thy- was 6.0 and 0.8%, respectively. For the octoploid cultivar Blackwell, it was 3.0 and 0.6% with LBA4404Thy- and EHA105Thy-, respectively (Table 2). The *Agrobacterium* strain LBA4404Thy- consistently outperformed the EHA105Thy- strain across the two switchgrass cultivars. In addition, the tetraploid cultivar Summer outperformed the octoploid cultivar Blackwell regardless of the *Agrobacterium* strains used for infection. This difference is especially pronounced when both were infected with LBA4404Thy-. When EHA105Thy- was used for infection, the overall transformation frequency was low for both cultivars and the difference between the cultivars were minimal (Table 2).

### The Effect of Optical Density of *Agrobacterium* on Transient Transformation

**FIGURE 6:**
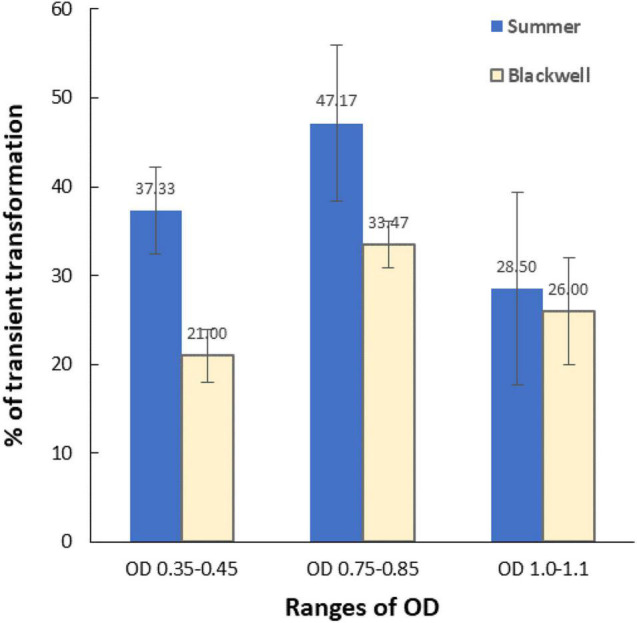
The effect of the optical density (OD_650_) of *Agrobacterium* culture (LBA4404Thy-) on transient transformation frequencies. X-axis, three ranges of OD; Y-axis, percent of immature leaf segments emitting GFP signals. Vertical bars associated with each data point is the standard deviation representing three experiments. Detection of GFP was carried out using a Zeiss fluorescence microscope 5–7 days after *Agrobacterium* infection.

To determine the optimum *Agrobacterium* OD for transformation, three OD_650_ ranges of LBA4404Thy- were tested for their effect on transient transformation frequency. Transient expression of GFP (Figure 2B) was assessed 5–7 days following co-cultivation by examining GFP signals under a Zeiss florescent microscope. Figure 6 shows the percentage of immature leaf segments emitting GFP for each cultivar with the three tested OD ranges. For Summer, the transient transformation frequency was 37.3% for the OD range of 0.35–0.45, 47.2% for OD range of 0.75–0.85, and 28.5% for OD range of 1.0–1.1. This indicated that OD of 0.75–0.85 outperformed the other two OD ranges based on GFP transient expression analysis. The Blackwell cultivar exhibited a similar trend with the OD range of 0.75–0.85 resulted in the highest transient GFP expression at 33.5% while the transient transformation frequency for OD of 0.35–0.45 and 1.0–1.1 were 21 and 26%, respectively.

## Discussion

Upland switchgrass is widely adapted to the North Central and Northeast of the United States with its rhizomatous growth habit and excellent winter hardiness. While genetic transformation is routinely performed for lowland switchgrass cultivars, upland switchgrass cultivars remain highly recalcitrant to genetic transformation mainly due to its inability to regenerate *in vitro* (Song et al., 2012; Merrick and Fei, 2015; Ogawa et al., 2016), creating a bottleneck for studying gene function using reverse genetics approach or rapid germplasm enhancement using genome editing technology. By overexpressing two morphogenic genes from maize, *ZmBbm* and *ZmWus2* in immature leaf segments, we established an efficient and reproducible genetic transformation protocol for two upland switchgrass cultivars, a tetraploid Summer, and an octoploid Blackwell, none of which has been transformed before. Our results also showed that the morphogenic transgenes *ZmBbm* and *ZmWus2* can be successfully excised from 40% of the transgenic plants of Summer and 33.3% of the transgenic plants of Blackwell with the Cre-Lox recombination system, eliminating the possible undesirable pleiotropic effects on plant growth and reproduction (Lowe et al., 2018). Taken together, our results clearly showed that overexpressing morphogenic genes can overcome transformation recalcitrance in upland switchgrass. Extensive omics resources have been developed for upland switchgrass (Palmer et al., 2015, 2017) and the transformation protocol developed in the present study will speed up genomics-related research.

Regeneration of switchgrass can be accomplished with a number of different types of explants including caryopsis (Li and Qu, 2011; Ogawa et al., 2016), immature inflorescence or young leaf segments (Denchev and Conger, 1994; Fei et al., 2000; Burris et al., 2009). Caryopsis has been the primary source of explant for lowland switchgrass transformation in the past due to its year-round availability (Li and Qu, 2011; Liu et al., 2018). When caryopses of upland switchgrass cultivars are used as explants, smooth, slow growing non-embryogenic calli were induced. These calli are referred to as type I callus and are often non-regenerative (Liu et al., 2015). At the time of preparing this manuscript, there are only two successful reports on transformation of upland switchgrass cultivars. Liu et al. (2015) identified the so called “shell-core” structure in type I calli and isolating the pre-embryogenic “core” from the “shell” resulted in the development of highly regenerative type II callus in two upland switchgrass cultivars, Blackwell and Dacotah. Further the authors reported a transformation frequency of 7.5% for Dacotah, a tetraploid upland cultivar while there was no mentioning of any success with the cultivar Blackwell. Ogawa et al. (2016) reported that by optimizing cocultivation and preculture conditions, improved transformation frequencies of lowland cultivars were obtained when type I calli were used for transformation. They also reported one of the three tested type I callus lines of an upland cultivar, Trailblazer produced transgenic plants at a frequency of 7.5% while failed to obtain any transgenic plants for the remaining callus lines of Trailblazer or the upland cultivar Blackwell. It is worth noting that explants used for *Agrobacterium*-mediated transformation in both studies were calli derived from caryopsis culture. Switchgrass cultivars are highly heterozygous and heterogeneous because they are open-pollinated synthetic cultivars (Casler, 2012). Therefore, each caryopsis represents a unique genotype. Once a caryopsis forms callus and regenerates plants, its identity can no longer be reproduced. In the present study, we used immature leaf segments taken from *in vitro*-grown seedlings which can be maintained indefinitely *via* tillering or *in vitro* subculture, giving year-round availability. If a highly responsive donor genotype is identified, the donor plant can be maintained indefinitely, thus providing a permanent superior source of explants for *Agrobacterium*-mediated transformation.

Despite the promise of using morphogenic genes in overcoming genotype dependency in genetic transformation, the choice of switchgrass cultivar appears to have a big influence on the transformation frequency. Regardless of the *Agrobacterium* strains used for infection, the tetraploid cultivar Summer outperformed Blackwell. As discussed earlier, neither Liu et al. (2015) nor Ogawa et al. (2016) successfully transformed Blackwell despite the former successfully transformed the upland cultivar Dacotah and made a significant improvement on regeneration in Blackwell. Despite a clear difference between the two cultivars on their responses to genetic transformation, genotype effect on the response to transformation within a cultivar cannot be determined because immature leaf segments were collected from genetically distinct individuals of each cultivar and were bulked for culture. Identifying genotypes within each cultivar with superior response to transformation can further improve transformation frequency. In addition, the tissue systems within a single grass leaf are highly heterogeneous with the presence of the intercalary meristems immediately below and above the ligule (Christians et al., 2016). Leaf bases containing meristematic regions have been used successfully in tissue culture of species from Poaceae (Haydu and Vasil, 1981; Hanning and Conger, 1982; Wernicke and Brettell, 1982). Identifying the age and location of leaves from which most transformation responsive immature leaf segments are harvested has the potential to further improve transformation efficiency.

The auxotrophic *Agrobacterium* strains rely on thymidine for growth and are thus highly advantageous because it allows for much better control of *Agrobacterium* overgrowth after the co-cultivation stage (Aliu et al., 2020). *Agrobacterium* will also less likely persist in callus or regenerated plants because of its dependence on thymidine for survival which is not supplemented in plant culture media. This also makes verification of the integration of the transgene less burdensome because possible false PCR positives are much less likely to occur. EHA105, along with AGL1 and C58C1 has traditionally been a choice of strain for *Agrobacterium*-mediated transformation in grasses including switchgrass (Wang and Ge, 2005; Kim et al., 2007; Xi et al., 2009). In the present study, however, we showed that EHA105 was much less efficient than the LBA4404 strain. The reason for this inconsistency is not clear, but we suspect the additional virulence genes brought in by the helper plasmid may have an undesirable impact on EHA105, a hypervirulent strain. Evaluation of three OD_650_ ranges on transient transformation suggested that the OD range 0.75–0.85 is optimum. However, further study is needed to determine if OD ranges can affect stable transformation frequency.

Using the *Bbm/Wus2* morphogenic genes, embryogenic callus could be readily formed from the leaf segment explants for the upland switchgrass. This rapid generation of embryogenic callus upon infection was not observed in previous transformation experiments using conventional standard *Agrobacterium* binary vectors, nor in a pilot experiment using *Agrobacterium* strain carrying only the helper plasmid PHP71539. The later observation suggests that culture media alone used in this work could not induce the callus formation.

While the morphogenic genes were effective in callus production, overexpression of the genes have been shown to exhibit undesirable pleiotropic effects on growth and development of the transgenic maize plants (Lowe et al., 2018). Abnormal shoot development was also observed in this study in GFP-expressing switchgrass calli that did not receive heat shock treatment. Excision of these genes eliminates the associated negative pleiotropic effect, allowing successful plant regeneration and normal plant development. The Cre-Lox recombination system has been successfully used to excise genes when the *cre* gene was driven by either a desiccation-inducible (Lowe et al., 2016) or a heat shock inducible promoter (Masters et al., 2020). In this study a maize heat shock protein gene promoter was used to induce the expression of the *cre* gene which resulted in an excision efficiency of 33 and 40%, respectively, for Blackwell and Summer. This removal frequency is comparable to previous reports in which a heat shock induced Cre-Lox excision system was used (Chong-Pérez et al., 2012; Wang et al., 2020).

One noteworthy information is that no selective agent was used in the protocol described in this work other than the GFP visual marker which was used to validate the transformation status of the calli. The morphogenic gene construct PHP93739 has a *ZmAls* gene cassette, which allows for herbicide ethametsulfuron (EMS) selection. Various EMS concentrations, ranged from 0.05 to 1.25 mg/L, were evaluated for their effects on the production of GFP-expressing callus. Our preliminary data showed that the percentages of immature leaf segments producing GFP-expressing embryogenic callus were severely reduced in both Summer and Blackwell on media containing EMS. No embryogenic callus formation was observed on medium containing EMS at 0.075 mg/L or above. While no selection was used, this work was benefited from the visual marker gene *gfp* on the transformation construct for effective screening. Every single plant regenerated from GFP-expressing callus was transgenic. It is worthwhile to carry out a more refined evaluation for EMS concentrations below 0.05 mg/L, for any construct that does not have a visual marker gene.

In conclusion, a reproducible genetic transformation protocol was established for two recalcitrant upland switchgrass cultivars, a tetraploid Summer and an octoploid Blackwell. The utilization of overexpression of morphogenic genes (*ZmBbm* and *ZmWus2*) is the key component for the success of this work. Using immature leaf segments derived from *in vitro* germinated seedlings and auxotrophic *Agrobacterium* strains, this protocol can produce embryogenic callus materials that were not possible for these two cultivars in the past. Following heat treatments of GFP-expressing embryogenic calli, *ZmBbm* and *ZmWus2* genes could be successfully removed, thus enabling the regeneration of transgenic plants. Our successful transformation of recalcitrant upland switchgrass enables gene function analysis or rapid germplasm enhancement *via* gene editing technology.

## Data Availability Statement

The raw data supporting the conclusions of this article will be made available by the authors, without undue reservation. Request of the construct PHP93739 and PHP71539, and *Agrobacterium* strain LBA4404Thy- should be directed to William Gordon-Kamm at Corteva Agriscience (William.gordon-kamm@corteva.com). Request of *Agrobacterium* strain EHA105Thy- should be directed to KW (kanwang@iastate.edu).

## Author Contributions

SF conceived the original research plans. NX conducted the experiments. MK assisted in data collection and presentation. JZ assisted in trouble-shooting process. NX, KW, and SF performed data analysis and prepared the manuscript. All authors contributed to discussion and revision of the manuscript.

## Conflict of Interest

The authors declare that the research was conducted in the absence of any commercial or financial relationships that could be construed as a potential conflict of interest.

## Publisher’s Note

All claims expressed in this article are solely those of the authors and do not necessarily represent those of their affiliated organizations, or those of the publisher, the editors and the reviewers. Any product that may be evaluated in this article, or claim that may be made by its manufacturer, is not guaranteed or endorsed by the publisher.
